# Datasets for the comparison of monthly spatiotemporal precipitation and temperature changes in a predicted GeoS-NCEP/NCAR-ANN dataset

**DOI:** 10.1016/j.dib.2023.109843

**Published:** 2023-11-26

**Authors:** Majid Javari

**Affiliations:** College of Social Science, PayameNoor University, PO Box 19395-3697, Tehran, Iran

**Keywords:** Temperature, Precipitation, Temporal, Spatial

## Abstract

This paper introduces the Precipitation and Temperature Database (PTDB), a comprehensive multi-database encompassing monthly precipitation and temperature values for about 10368 precipitation points and 65535 temperature points worldwide. The extraction of this database was facilitated by employing a large-scale GeoS-NCEP/NCAR-ANN, which operates within a global coordinate system ranging from 90°NS latitude to 180° EW longitude. The data used for this extraction was derived from a 30-year climate data set from 1991 to 2020. The PTDB is a valuable resource for conducting spatiotemporal data analysis about large-scale climate change. It is readily accessible for download through the Mendeley data platform. This distribution includes the monthly interpolated data for all 10368 precipitation points and 65535 temperature points. The geostatistical methods used in ArcGIS Pro3, along with the artificial neural network (ANN) started in MATLAB, were used to generate global monthly forecast data sets. These data sets have a resolution of 14361.49 km^2^ for precipitation and 2272.068 km^2^ for temperature. The extracted monthly raster data, converted to Excel format, can prove instrumental in climate change modeling, forecasting, and the assessment of natural hazards.

Specifications Table

**Table:** 

Subject	Climate change patterns; climatology and climate Planetary variability
Specific subject area	This research investigation focused on the database of accurate monthly temperature and precipitation
Data format	Raw Predicted and Designed data
Type of data	The geodatabase comprises monthly precipitation and temperature data, along with supplementary information, meticulously organized into 10368 data points for precipitation and 65535 data points for temperature.
Data collection	The initial layer of precipitation and temperature was constructed in an AFR format, comprising a 72 × 144 matrix with a pixel depth of 16 bits. This layer was generated at regular time intervals of 28.5 days, encompassing the period from January 1, 1991, to December 1, 2020. The primary layer underwent consistent updates every 5 minutes. This layer is documented in the NCEP-NCAR Reanalysis1, which incorporates the NCEP/Climate Prediction Center. Projected data for precipitation and temperature were produced as a geodatabase, using a matrix size of 10368 × 12 for precipitation and 65535 × 12 in various formats such as Excel format.
Data source location	The term “distributed datasets” pertains to a dataset that encompasses 10368 temperature points and 65535 precipitation points, each with specific coordinates, covering various regions of the world, including Asia, Africa, North America, South America, Antarctica, Europe, and Australia. This dataset is based on a 30-year climate data set and was predicted using geostatistical and statistical methods to determine its spatiotemporal course. To analyze the spatiotemporal variability of precipitation and temperature, comparisons were made between predicted data errors from NCEP/NCAR reanalyzed data using different patterns, using ArcGIS Pro3 and an artificial neural network (ANN) in MATLAB. https://data.mendeley.com/datasets/9yjb77wxbz Topic sample collection: NCEP/NCAR reanalyzed data Extracted data using ArcGIS Pro3: https://data.mendeley.com/datasets/9yjb77wxbz/4 Predicted data using Geostatistical models **Geodatabase:** https://data.mendeley.com/datasets/dmkjnk94xc
Data accessibility	Predicted datasets (GeoS-NCEP/NCAR-ANN data) describe a set of 10368 points from temperature and 65535 points from precipitation elements with specific coordinates. Repository name: Datasets for comparison of spatiotemporal changes in precipitation and temperature in an extracted set of GeoS-NCEP/NCAR-ANN data Data identification number: DOI:10.17632/9yjb77wxbz.4 Direct URL to data: https://data.mendeley.com/datasets/9yjb77wxbz/4 **Direct URL to geodatabase: DOI:**10.17632/dmkjnk94xc.2. https://data.mendeley.com/datasets/dmkjnk94xc
Related research articles	Comparing causal techniques for rainfall variability analysis using causality algorithms in Iran doi:10.1016/j.heliyon.2018. e00774

## 1 Value of the Data

The PTDB database offers access to monthly temperature and precipitation datasets, enabling the assessment and comparison of spatiotemporal fluctuations in these climatic variables. These datasets have been conveniently provided in an Excel format, facilitating their utilization. Researchers with a vested interest in global temperature and precipitation variability stand to gain significant advantages from the use of these data. Geostatistical and statistical techniques can investigate temperature and precipitation variability, as well as climatic conditions, on a global scale. The PTDB, comprising monthly temperature and precipitation datasets, can be effectively used for the evaluation and comparison of spatiotemporal changes in precipitation and temperature, in a user-friendly Excel format. This valuable resource will benefit researchers exploring variations in temperature and precipitation on a global scale. The data can be effectively used with geostatistical and statistical methods to examine temperature and precipitation variability, as well as climatic conditions, worldwide.

• The dataset extracted and predicted (monthly data) of PTDB and the predicted monthly precipitation and temperature datasets can be used for assessments in climatic change modeling, climate change predictions, and climate natural dangers evaluations;
• The predicted global monthly precipitation and temperature data can be used for simulating monthly global hydroclimatic processes;
• The predicted global monthly precipitation and temperature data can optimize the location of climate-related activities worldwide;
• The predicted global monthly precipitation and temperature data can identify droughts and climate-related events worldwide.

Monthly temperature and precipitation data records are imperative in academic research. Although there are several global datasets available, only a limited number offer temperature and point precipitation in a suitable format. These datasets differ significantly in terms of temporal and spatial resolution, predicted error, and standard distribution. One such dataset is the PTDB, also known as GeoS-NCEP/NCAR-ANN data, and the ArcGIS geodatabase of GeoS-NCEP/NCAR-ANN, which is an integral part of the World Climate Research (WCR) initiative. The primary objective of the WCR is to enhance our comprehension of the intricate interactions between natural patterns and climate-related phenomena, specifically focusing on optimizing sites for climate-related activities. However, the PTDB is predominantly used by researchers to estimate climate science from global climate events. Assessing climate conditions based on monthly temperature and precipitation data with appropriate spatiotemporal resolution poses a challenging task due to the uneven distribution of data. To tackle this issue, we have compiled a comprehensive global dataset comprising nearly 10368 temperature points and 65535 precipitation points. The predicted monthly temperature and precipitation data are stored in Mendeley Data and can be accessed through the GeoS-NCEP/NCAR-ANN dataset. Also, we have employed the Artificial Neural Network Toolbox in MATLAB to calculate the monthly temperature and precipitation data, enabling a more accurate assessment of the spatiotemporal variability of climate patterns.

## 2 Data Description

**Fig. 1:**
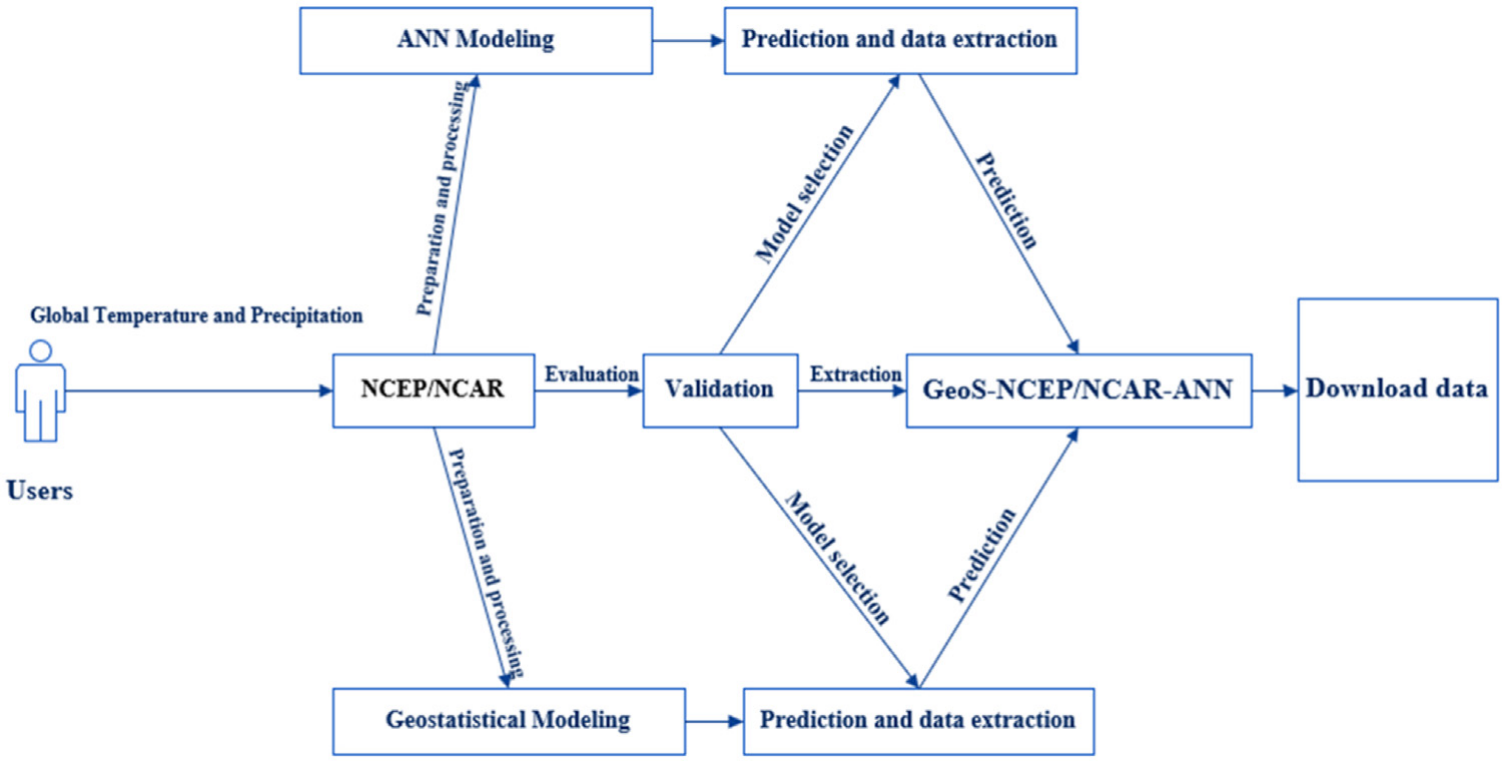
The design stages of predicted global temperature and precipitation (PTDB) in the first phase.

**Fig. 2:**
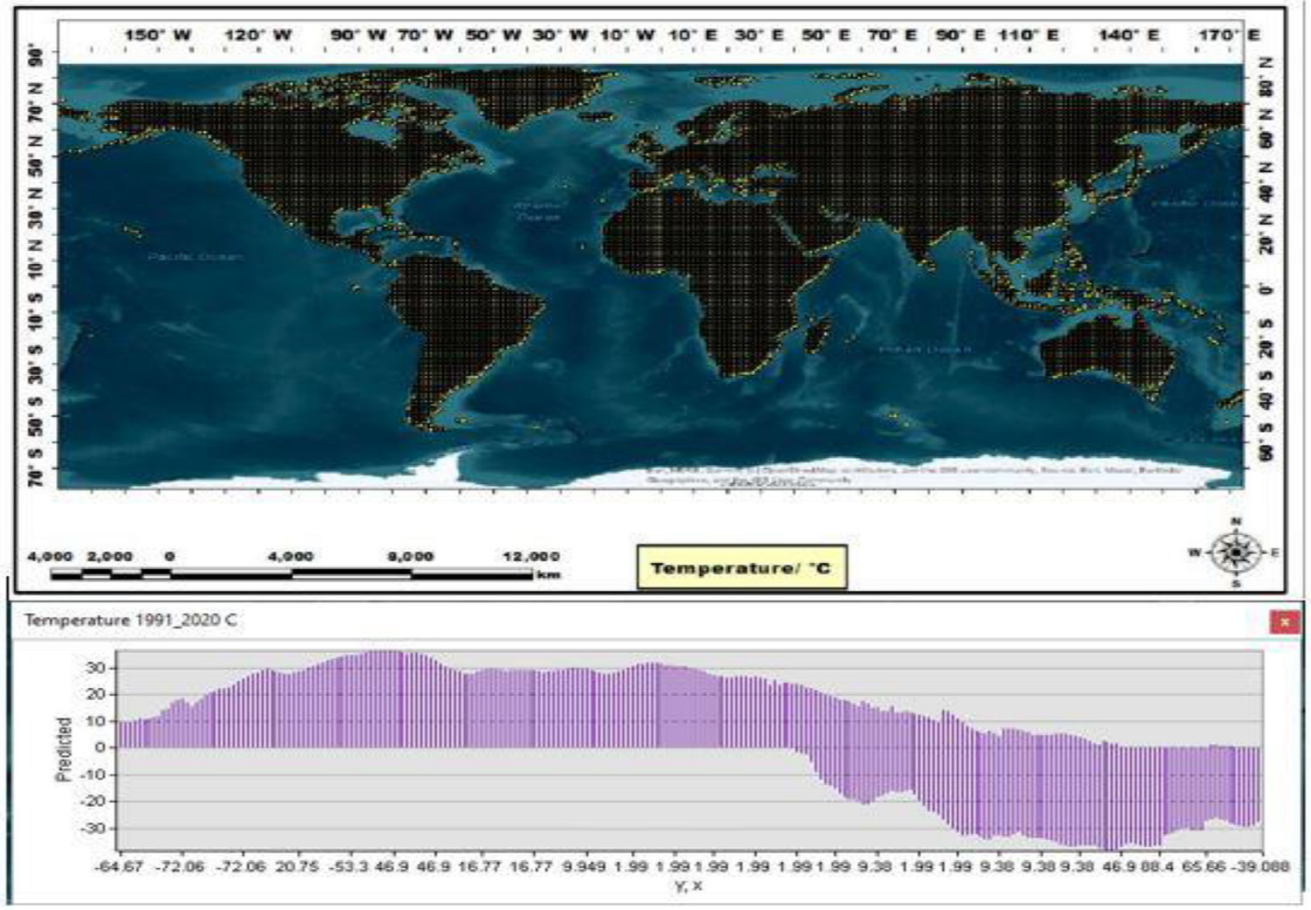
Point distribution of temperature worldwide.

**Fig. 3:**
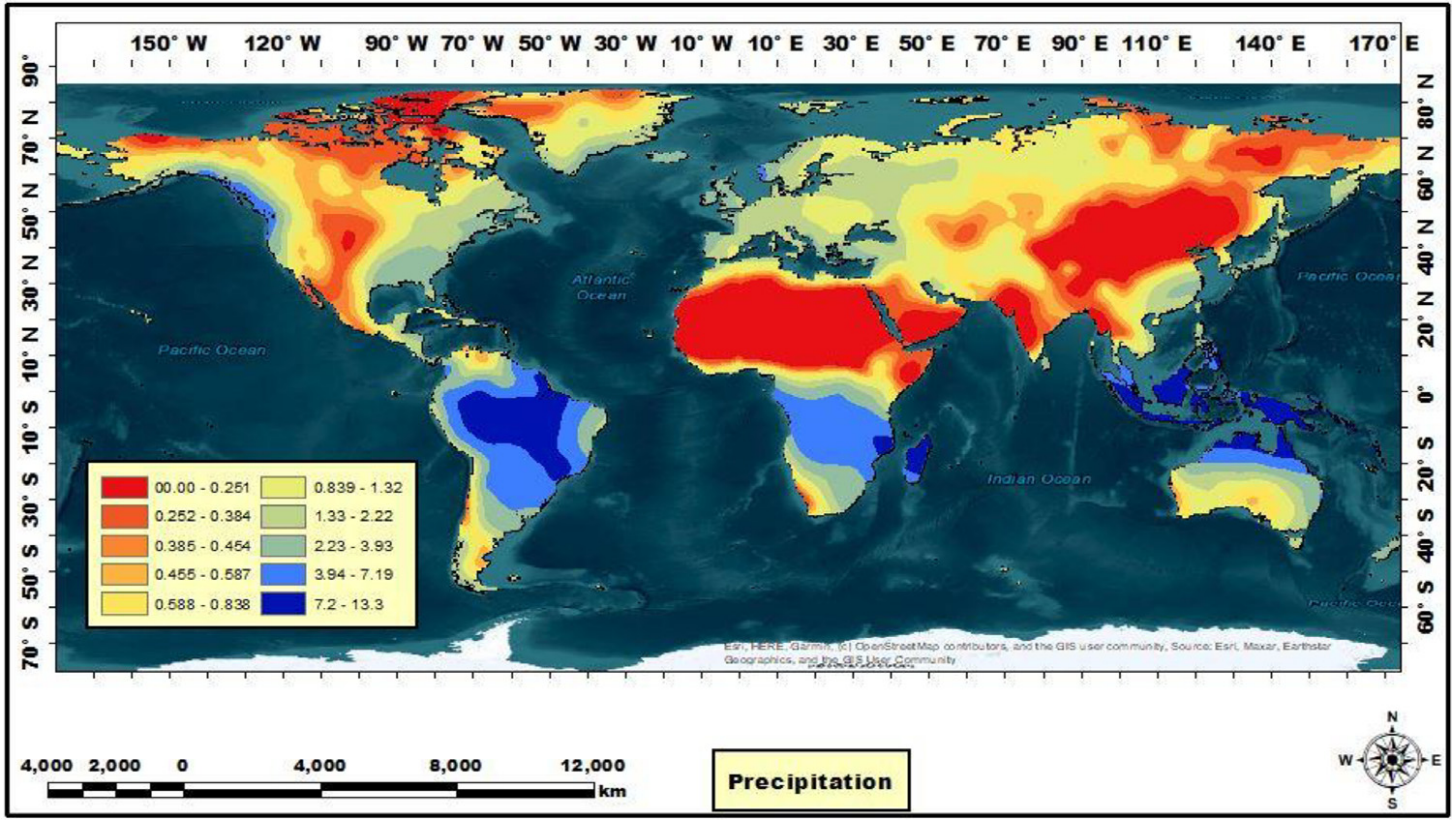
Distribution of precipitation worldwide.

**Fig. 4:**
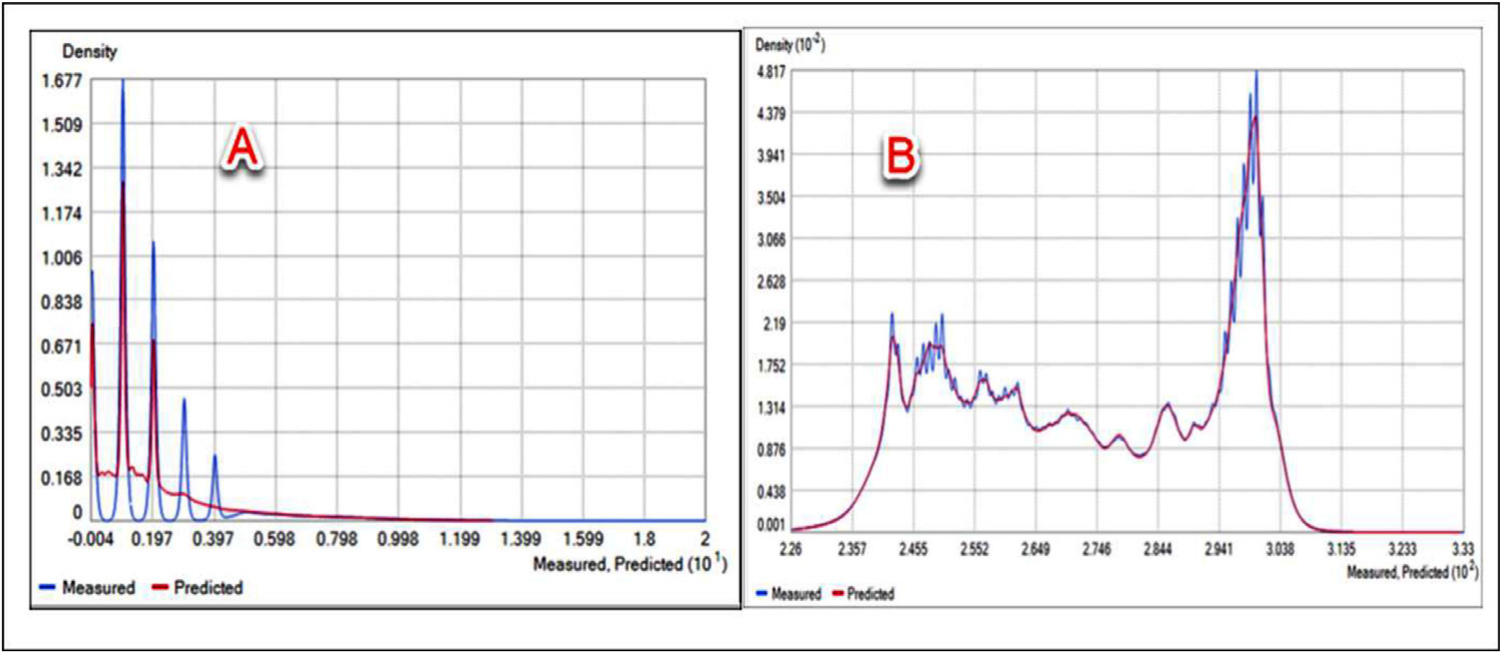
Density of predicted precipitation (A) and temperature (B).

To facilitate a comparative analysis of global spatiotemporal temperature and precipitation variability, a monthly compilation of temperature and precipitation data was integrated into the Mendeley Data platform. Then an artificial neural network (ANN) using the Levenberg-Marquardt algorithm was used to predict global data, using the practical estimation of the monthly temperature and precipitation database and forecast data. The resulting predicted data, called PTDB, encompassed information from 10368 temperature points and 65535 precipitation points, which were geographically distributed globally. Geostatistical methods were then applied to the PTDB, using ArcGIS Pro3 to forecast monthly temperature and precipitation values, and MATLAB software to estimate monthly temperature and precipitation values. This approach helped with a novel representation of global spatiotemporal temperature and precipitation data. All predicted temperature and precipitation data had been categorized and are readily accessible for download on the Mendeley platform, under the dataset name “GeoS-NCEP/NCAR-ANN dataset and ArcGIS geodatabase of GeoS-NCEP/NCAR-ANN” (Fig. 1). The ArcGIS geodatabase of GeoS-NCEP/NCAR-ANN is a comprehensive and sophisticated tool used in temperature and precipitation analysis. This database serves as a repository for the GeoS-NCEP/NCAR-ANN dataset, a valuable resource for researchers and scholars in various academic disciplines. Using ArcGIS technology enhances the accessibility and usability of this geodatabase, allowing for efficient data management and analysis. Integrating this geodatabase into academic research on temperature and precipitation facilitates the exploration and understanding of complex climatic phenomena, contributing to the advancement of knowledge in the field. This geodatabase offers a comprehensive information model that helps with the representation and management of geographic information to analyze monthly temperature and precipitation patterns. This information model was made through monthly temperature and precipitation feature classes, along with their associated attributes. Advanced databases practically solve maintaining spatial integrity and managing spatial relationships within the context of monthly temperature and precipitation data. This database encompasses the temperature and precipitation layer as a dBASE_Table, the temperature and precipitation points represented as a feature class, and the distribution of temperature and precipitation changes depicted as a raster layer on a global scale. The Mendeley dataset was used to organize monthly data, specifically temperature (abbreviated as “Tem”) and precipitation (abbreviated as “Precip”), to construct a monthly distribution of temperature and precipitation for developing the PTDB. Then the PTDB was employed to distribute databases for various regions, including Asia, Africa, North America, South America, Antarctica, Europe, and Australia. These databases were formatted differently and had 10368 temperature points and 65535 precipitation points (Fig. 2). However, the distributed datasets, called GeoS-NCEP/NCAR-ANN data and ArcGIS geodatabase of GeoS-NCEP/NCAR-ANN, encompassed a set of distributed points with specific features calculated for all global regions based on a 30-year climate dataset (Fig. 3). Temporal data and spatially predicted data for global temperature and precipitation were defined using data reanalyzed by NCEP/NCAR and data extracted by ArcGIS Pro3 and MATLAB software, covering the reference period of 1991-2020. Geostatistical and statistical methods were used to predict spatiotemporal variability and examine the regionalization of temperature and precipitation changes worldwide [1]. Temporal and predicted world temperature and precipitation datasets have been generated based on reanalyzed data from NCEP/NCAR and extracted data from ArcGIS Pro3 for the reference period of 1991-2020 (Fig. 4). Monthly temperature and precipitation predictions were incorporated into the PTDB through an artificial neural network (ANN), with calculations performed on 10368 temperature points and 65535 precipitation points. Also, the PTDB database includes supplementary data to aid in spatiotemporal analysis, such as Error, XY coordinates, StdError, and Stdd Error. Geostatistical methods, specifically ordinary kriging, were employed as an effective spatial technique for regionalizing point data into climatologically predicted regions [2]. The present study uses high-resolution temperature data, with a resolution of 0.5 × 0.5, and global coverage from 1991 to 2020. The data was extracted from reanalyzed NCEP/NCAR data [3], available at https://psl.noaa.gov/data/gridded/index.html. The NCEP/NCAR temperature observations were reanalyzed based on monthly series, as presented in Table 1. Each monthly database comprises ten folders, including Source_ID, which provides the layers with their coordinates; Measured, which stores primary data expressed in temperature and precipitation, measured in degrees Kelvin and millimeters per day, respectively; Predicted, which stores predicted data expressed in temperature and precipitation, measured in degrees Kelvin and millimeters per day, respectively; Error, which stores temperature and precipitation data errors, calculated as the difference between the predicted and validation field values; StdError, which stores standardized errors for temperature and precipitation data; Stdd_Error, which stores standardized errors for temperature and precipitation data; and NormValue, which stores distribution values for temperature and precipitation data, expressed as the normal distribution value (x-axis) corresponding to the standardized prediction errors (y-axis) in the normal QQ chart. Also, Table 2 presents the NCEP/NCAR precipitation datasets, which were reanalyzed based on monthly series, as presented in Table 3. The monthly dataset comprises two files containing monthly averaged precipitation rate values. These values are obtained from five distinct types of satellite estimates, GPI, OPI, SSM/I scatter, SSM/I emission, and MSU, in addition to gauge data. To capture the spatial variability of temperature and precipitation, an empirical semivariogram was employed as the spherical model. It is expected to use the spherical model with the variability index as an alternative spatial structure [4]. The present study involved an monthly temperature series derived from reanalyzed NCEP/NCAR data from 1991 to 2020. The empirical semivariogram values were grouped and averaged, and the resulting data were evaluated as controls to visualize the described data. The clustered points were estimated over 30 years to analyze the local variation in the data. Mean values were also calculated to fit model-described data using spatial autocorrelation. The study assessed a strong negative spatial correlation between datasets and latitude using the nugget, range, partial sill, and anisotropy values. During the evaluation, the selected model was found to affect the prediction of the data using both known and unknown values, with the influence of the model on the datasets being calculated using nearest neighbors. Based on the 30 years, the semivariogram modeling data was estimated and measured using structural analysis or variography. The errors or spatial distribution of the variances at distances smaller than the sampling interval were estimated based on the nugget effect. Also, the predicted spatial variation of the data was calculated, considering the scaling of the spatial variation. The spatial variations revealed the data distribution based on precipitation (10368 points) and temperature (65535 points) and allowed for an estimation of the differences in data distribution based on spatiotemporal patterns. The data helped with a spatial-temporal comparison [5] of the differences in data distribution on different scales [6]. Finally, the Make Multidimensional Raster Layer tool was used to extract a subset of data and discover the multidimensional raster dataset based on temporal and spatial patterns.

**Table 1:** Description of the extracted and predicted data.

Data name	Data description
January temperature	The dataset used to measure temperature changes in January
February temperature	The dataset used to measure temperature changes in February
March temperature	The dataset used to measure temperature changes in March
April temperature	The dataset used to measure temperature changes in April
May temperature	The dataset used to measure temperature changes in May
June temperature	The dataset used to measure temperature changes in June
July temperature	The dataset used to measure temperature changes in July
August temperature	The dataset used to measure temperature changes in August
September temperature	The dataset used to measure temperature changes in September
October temperature	The dataset used to measure temperature changes in October
November temperature	The dataset used to measure temperature changes in November
December temperature	The dataset used to measure temperature changes in December
January precipitation	The dataset used to compute precipitation changes in January
February precipitation	The dataset used to compute precipitation changes in February
March precipitation	The dataset used to compute precipitation changes in March
April precipitation	The dataset used to compute precipitation changes in April
May precipitation	The dataset used to compute precipitation changes in May
June precipitation	The dataset used to compute precipitation changes in June
July precipitation	The dataset used to compute precipitation changes in July
August precipitation	The dataset used to compute precipitation changes in August
September precipitation	The dataset used to compute precipitation changes in September
October precipitation	The dataset used to compute precipitation changes in October
November precipitation	The dataset used to compute precipitation changes in November
December precipitation	The dataset used to compute precipitation changes in December

**Table 2:** Description of the database of temperature.

Measured_Tem°K	Predicted_Tem°K	Error	StdError	Stdd_Error	NormValue	Source_ID	POINT_X	POINT_Y	Tem°C
243.00	277.52	34.52	25.48	1.36	2.22	0	-40.75	83.75	-30.15
243.00	242.27	-0.73	1.97	-0.37	-1.17	1	-40.25	83.75	-30.15
243.00	242.97	-0.03	1.77	-0.02	-0.08	2	-39.75	83.75	-30.15
243.00	243.02	0.02	1.77	0.01	0.10	3	-39.25	83.75	-30.15
243.00	243.05	0.05	1.77	0.03	0.18	4	-38.75	83.75	-30.15
243.00	243.04	0.04	1.77	0.02	0.15	5	-38.25	83.75	-30.15
243.00	243.03	0.03	1.77	0.02	0.13	6	-37.75	83.75	-30.15
243.00	242.98	-0.02	1.77	-0.01	-0.04	7	-37.25	83.75	-30.15
243.00	242.95	-0.05	1.77	-0.03	-0.14	8	-36.75	83.75	-30.15
243.00	242.97	-0.03	1.77	-0.02	-0.08	9	-36.25	83.75	-30.15
243.00	243.29	0.29	1.77	0.16	0.60	10	-35.75	83.75	-30.15
⋮	⋮	⋮	⋮	⋮	⋮	**65535**	⋮	⋮	⋮

**Table 3:** Description of the database of precipitation.

Measured mm/day	Predicted mm/day	Error	StdError	Stdd_Error	NormValue	Source_ID	POINT_X	POINT_Y
1	0.656	-0.344	0.989	-0.348	-1.048	0	136.250	48.750
1	1.056	0.056	0.989	0.056	0.375	1	138.750	48.750
1	1.268	0.268	0.989	0.271	0.797	2	141.250	48.750
1	1.519	0.519	0.989	0.525	1.370	3	143.750	48.750
2	1.570	-0.430	0.989	-0.435	-1.207	4	146.250	48.750
2	1.974	-0.026	0.989	-0.026	-0.533	5	148.750	48.750
2	2.165	0.165	0.989	0.167	0.581	6	151.250	48.750
2	2.102	0.102	0.989	0.104	0.498	7	153.750	48.750
2	2.341	0.341	0.989	0.345	0.985	8	156.250	48.750
3	2.596	-0.404	0.989	-0.409	-1.142	9	158.750	48.750
3.	2.826	-0.174	0.989	-0.176	-0.738	10	161.250	48.750
3	2.838	-0.162	0.989	-0.164	-0.718	11	163.750	48.750
⋮	⋮	⋮	⋮	⋮	⋮	**10368**	⋮	⋮

## 3 Experimental Design, Materials and Methods

In this study, spatiotemporal data were used to explore global temperature and rainfall patterns over the reference period from 1991 to 2020. Using ArcGIS Pro 3, the Reanalysis layer of NCEP_NCAR was used and extracted. To collect spatiotemporal data and regionalize temperature and precipitation changes worldwide, geostatistical and statistical techniques have been applied. Then, a new database was created using ArcGIS Pro3 to systematically analyze surface temperature and precipitation data at 90°NS latitude and 180°EW longitude. In the following stage, 10368 pixels/degree for precipitation and 65535 pixels/degree for temperature were extracted from the NCEP-NCAR Reanalysis. The initial phase of this section involved the retrieval of raw precipitation and temperature layers from NCEP-NCAR Reanalysis in NetCDF (network common data form) format. NetCDF is a machine-independent format designed for the representation of scientific data. The subsequent stage entailed optimizing raster information in an enterprise geodatabase raster data type, using multidimensional information with stdtime 906 to achieve ideal raster information with 73 × 144 matrix values and 32-bit pixel depth. The layers in the Spheroid WGS 1984 geographic system coordinate were used to develop global monthly point layers. Preparing geodatabase raster data involved extracting precipitation and temperature layers as point layers. The calculation of precipitation and temperature layers was based on geostatistical models, which first estimated the point layer of precipitation and temperature. Then, the coordinated precipitation and temperature in Excel format and monthly distribution were calculated. To obtain enterprise geodatabase raster data, the Make Multidimensional Raster Layer tool in ArcGIS Pro3 was used. This tool helps create a multidimensional raster layer from a multidimensional raster dataset by extracting a subset of variables. The multidimensional raster layer was generated using the cloud raster format (CRF) derived from netCDF files. The dimension definition parameter (DDP) was used to slice dimensions based on 12 months of precipitation and temperature. Different dimension definition options were used for distinct scenarios. First, the full range for each dimension was used for the 75 years under consideration. Second, precipitation and temperature data for the months over the 75 years were extracted. The values option was selected, with the Dimension set to StdTime and the Values set to January to December was selected. A distinct framework was used to systematically present temperature and precipitation data based on sector type 4 and 45 degrees, semimajor axis and semiminor axis (73.09), number of lags (12), lag size (8.89), and neighborhood search (standard). Also, the partial sill (7.93) and (142.75) were selected. Geostatistical methods, specifically Ordinary Kriging, were an effective spatial technique to regionalize point data into climatologically predicted regions. The regionalization of temperature and precipitation points as continuous in space requires detrending surface, semivariogram models [7], and search neighborhoods to identify temperature and precipitation zones from predicted regions as spatial variability patterns. Statistical methods were employed as an effective temporal technique to analyze the data into climatologically predicted changes [8]. The regionalization of temporal data from temperature and precipitation data discontinuous in space was achieved using descriptive methods such as mean, standard deviation, range, etc., and the examination of patterns in the variability of temperature and precipitation from recorded data as temporal variability patterns. The available data comprise spatiotemporally layered point raster with coordinate format. Using the Raster to Point tool, it is possible to use the data as a pixel series in specific dimensions to examine the variability of global temperature and precipitation [9,10]. A point in the data set with an attribute is generated for each cell of the input raster data set. The pixels at the center of the cells represent a specific quantity of temperature and precipitation. The pixel data have been transformed to reflect the geographical distribution of various regions across the globe and the underlying distribution of temperature and precipitation levels. The Add XY Coordinates tool has been used to convert these data. The goal of this study is to provide empirical evidence that will help with the computation of alterations in temperature and precipitation and evaluate their variability. The temperature and precipitation datasets were obtained from recently scrutinized NCEP/NCAR data. The findings of this investigation are as follows. The temperature and precipitation points, latitude and longitude, data area, data distribution, data variability, data gradient, data distribution pattern, and data predictability were extracted for each monthly database. The present study employed the Calculate Geometry Attributes (Data Management) tool, which is based on the Add XY Coordinate System tool in ArcGIS Pro3, to determine the locations and distribution of the data. The data was subsequently transformed into monthly Excel files through the conversion of each layer. The temperature and precipitation data points were extracted as raster cells utilizing the Raster to Point (Conversion) tool in ArcGIS Pro3, which employed data interpolation techniques. To assess the variability of the data, a comprehensive linear pattern of spatial coordinates was established for each cell within the input raster dataset, particularly within the temperature layers. A global database was established for this study, encompassing monthly temperature and precipitation patterns across the Earth's surface [11]. Kriging methods, which rely on autocorrelation, were employed for data preparation, with the autocorrelation basis being a function of distance. Ordinary kriging also incorporates a linear function of its spatial coordinates. Before selecting the most suitable interpolation model for decision-making, it is imperative to evaluate the methods using accuracy indicators. The accuracy and reliability of the methods were assessed through the utilization of error indicators, which quantify the disparity between predicted and measured values (predicted minus measured). During the data preparation phase, the distribution of positive and negative error values is considered. A positive error denotes a prediction that exceeds the measured value, while a negative error indicates a prediction that falls short of the measured value. The standard error is the predicted value derived from normal errors. The standardized error is obtained by dividing the error by the standard error. The mean error (ME) is the average of the cross-validation errors, and it should ideally be as close to zero as possible. A positive mean error suggests a tendency to overpredict values, while a negative mean error indicates a tendency to underestimate measured values. The Root Mean Square Error (RMSE) was estimated as the square root of the mean square prediction errors, and it measures the accuracy of predictions and estimates the deviation of predicted values from measured values. The mean standardized error (MSE) was calculated as the average of the standardized errors, and it measures model bias on a standardized scale based on comparable datasets with different values and units. The average standard error (ASE) was obtained by taking the root mean square of the standard errors. The present statistic evaluates the precision of a model by analyzing distributions closely centered on the predicted value. The root mean square of the standardized errors was computed as the root mean square standardized error (RMSSE). This metric assesses the accuracy of the standard errors by comparing the variability of the cross-validation errors to the estimated standard errors. Values below one indicate that the estimated standard errors are large, while values above one suggest that they are too small. The cross-validation indices were evaluated at a 90% confidence level. This statistic determines whether the standard errors conform to the predicted values. Values exceeding 90 indicate that the standard errors are disproportionately large relative to the predicted values, but values below 90 indicate that the standard errors are too small.

## Data Limitations

In the presented work, limited techniques were used to analyze climate data in temporal and spatial pattern format separately based on the gridded data. However, many possibilities for future models use multiple climate patterns. This would not allow the extraction of datasets for different models, which would be a helpful addition. The other major limitation of the PTDB is the multi-software design of the GeoS-NCEP/NCAR-ANN.

## Data Availability

The ArcGIS geodatabase of GeoS-NCEP/NCAR-ANN (Original data) (Mendeley Data) Datasets of GeoS-NCEP/NCAR -ANN (Original data) (Mendeley Data)
